# Negative correlation of cortical thickness with the severity and duration of abdominal pain in Asian women with irritable bowel syndrome

**DOI:** 10.1371/journal.pone.0183960

**Published:** 2017-08-31

**Authors:** Chian Sem Chua, Chyi-Huey Bai, Chen-Yu Shiao, Chien-Yeh Hsu, Chiao-Wen Cheng, Kuo-Ching Yang, Hung-Wen Chiu, Jung-Lung Hsu

**Affiliations:** 1 Graduate Institute of Biomedical Informatics, Taipei Medical University, Taipei, Taiwan; 2 Division of Gastroenterology, Department of Internal Medicine, Shin Kong Wu Ho-Su Memorial Hospital, Taipei, Taiwan; 3 Western Medicine Division, Hospital Lam Wah Ee, Penang, Malaysia; 4 Department of Public Health, School of Medicine, College of Medicine, Taipei Medical University, Taipei, Taiwan; 5 School of Public Health, College of Public Health and Nutrition, Taipei Medical University, Taipei, Taiwan; 6 Department of Diagnostic Radiology, Shin-Kong Wu Ho-Su Memorial Hospital, Taipei, Taiwan; 7 Department of Information Management, National Taipei University of Nursing and Health Sciences, Taipei, Taiwan; 8 Master Program in Global Health and Development, College of Public Health and Nutrition, Taipei Medical University, Taipei, Taiwan; 9 Department of Transportation & Logistics Management, National Chiao Tung University, Taipei, Taiwan; 10 School of Medicine, Taipei Medical University, Taipei, Taiwan; 11 Department of Neurology, Chang Gung Memorial Hospital Linkou Medical Center and College of Medicine, Chang-Gung University, Taoyuan, Taiwan; 12 Graduate Institute of Humanities in Medicine, Taipei Medical University, Taipei, Taiwan; Banner Alzheimer's Institute, UNITED STATES

## Abstract

**Background & aims:**

Irritable bowel syndrome (IBS) manifests as chronic abdominal pain. One pathophysiological theory states that the brain–gut axis is responsible for pain control in the intestine. Although several studies have discussed the structural changes in the brain of IBS patients, most of these studies have been conducted in Western populations. Different cultures and sexes experience different pain sensations and have different pain responses. Accordingly, we aimed to identify the specific changes in the cortical thickness of Asian women with IBS and to compare these data to those of non-Asian women with IBS.

**Methods:**

Thirty Asian female IBS patients (IBS group) and 39 healthy individuals (control group) were included in this study. Brain structural magnetic resonance imaging was performed. We used FreeSurfer to analyze the differences in the cortical thickness and their correlations with patient characteristics.

**Results:**

The left cuneus, left rostral middle frontal cortex, left supramarginal cortex, right caudal anterior cingulate cortex, and bilateral insula exhibited cortical thinning in the IBS group compared with those in the controls. Furthermore, the brain cortical thickness correlated negatively the severity as well as duration of abdominal pain.

**Conclusions:**

Some of our findings differ from those of Western studies. In our study, all of the significant brain regions in the IBS group exhibited cortical thinning compared with those in the controls. The differences in cortical thickness between the IBS patients and controls may provide useful information to facilitate regulating abdominal pain in IBS patients. These findings offer insights into the association of different cultures and sexes with differences in cortical thinning in patients with IBS.

## Introduction

Irritable bowel syndrome (IBS) is a chronic functional gastrointestinal disease. Patients with IBS frequently suffer from chronic abdominal pain and bloating. IBS affects 10%–20% of the global population and is more common in Western countries, affecting 7%–15% of the population in the United States [1]. IBS affects 6%–11.5% of the population in various Asian countries [2]. Among IBS patients, women have a 38% higher prevalence of constipation-predominant IBS [3]. Most IBS patients are aged between 30 and 50 years [4]. In Taiwan, the prevalence of functional gastrointestinal disorder is 26.2%, and 4.4% of these patients fulfill the Rome III criteria [5]. IBS patients in Taiwan are younger than those in other Asian countries and are predominantly female. Several diseases, including major depression, anxiety [6], fibromyalgia, chronic fatigue syndrome, and chronic pelvic pain, have been associated with IBS [7]. Several clinical criteria, more commonly the ROME III criteria [8], are used to diagnose IBS.

The brain–gut axis regulates the food digestion process in humans. However, this axis is also related to stress, mood disorder, abdominal pain, and altered bowel motility [9]. A change in the perceptual and reflex responses of the brain–gut axis is one of the hypothesis for explaining the pathophysiology of IBS. In functional magnetic resonance imaging (MRI) studies using rectal inflation stimulation in IBS patients, the brain regions for visceral sensation, emotion arousal, and attention processes [10] were more specifically activated. In a structural brain imaging study, brain structural imaging changes were observed in regions involving the anterior midcingulate cortex, dorsolateral prefrontal cortex, and insula, and these changes were related to the affective and interoperative processing and modulation of pain sensation and pain catastrophizing [11].

However, several factors can influence our sense and reaction to pain awareness, including cultural factors and gender [12]. A study revealed that Eastern populations have better pain tolerance than do Western populations, and women are more tolerant of pain than are men [13]. Given that numerous studies on IBS have been conducted on Western patients and that many of them have reported that different parts of the brain undergo structural changes, we proposed that in patients of different ethnicity and sex, differences in brain structural changes would be observable; accordingly, we conducted this study to compare IBS patients between Asian and Western populations. Our study focused on Asian Chinese women with IBS to minimize bias and to elucidate the differences in brain structural changes. We analyzed the relationship between abdominal pain due to IBS and cortical structural changes in a sample of East Asian female patients.

## Methods

This case–control study was conducted from January 01 to December 31, 2013, at Shin Kong Wu Ho-Su Memorial Hospital in Taipei City, Taiwan. The study was registered at ClinicalTrials.gov (NCT02179905). The study was approved by the Institutional Review Board of Shin Kong Wu Ho-Su Memorial Hospital (Serial number: 20120813R).

### Patient inclusion

We recruited 30 IBS patients (IBS group) and 39 healthy individuals (control group) from the Division of Gastroenterology at Shin Kong Wu Ho-Su Memorial Hospital. The inclusion criteria were being female and having received an IBS diagnosis according to the Rome III criteria. The patients were aged between 20 and 50 years old, and all of the patients were predominantly right handed-predominant. The inclusion criteria for the controls were being healthy and free of chronic abdominal pain. We excluded those who had colon or small intestinal diseases, psychiatric disease, or diabetes; those who received major surgery in the past 5 years; and those with metal implants. All participants were required to sign an informed consent form, which was approved by the Research Ethics Board. Finally, one patient was excluded due to an inability to undergo MRI scanning (n = 1); thus, 29 IBS patients included in the analysis.

### Questionnaires

The age and demographic data, including height, weight, and waist circumference, were recorded. All patients received a psychometric interview. Questionnaires for anxiety and depression were employed, and the 24-item Hamilton Depression Rating Scale [14] was used. The intensity of abdominal pain was scored using the Wong-Baker FACES Pain Rating Scale [15].

### MRI

Whole brain MRI was performed at Shin Kong Wu Ho-Su Memorial Hospital (Philips, 3T Achieva, Netherlands). Three scans were acquired: (1) transaxial T2-weighted scans [repetition time/echo time (TR/TE) = 3442/90 ms, number of excitations (NEX) = 2, voxel size = 0.23 × 0.23 × 5 mm], (2) FLAIR images (TR/TE = 8000/120 ms, inversion time = 2400 ms, NEX = 2, voxel size = 0.17 × 0.17 × 5 mm), and (3) high-resolution sagittal T1-weighted images (TR/TE = 7.3/3.5 ms, NEX = 1, voxel size = 1.0 × 1.0 × 1.0 mm). Patients had to remain immobile and calm during the scanning to prevent confounding results.

### FreeSurfer

Cortical thickness analysis was performed using FreeSurfer version 5.3.0. (Massachusetts General Hospital, Harvard Medical School; http://surfer.nmr.mgh.harvard.edu/), which is a tool with surface-based analysis functionality that can be used to analyze the three-dimensional views of a brain image. In the FreeSurfer, we used T1-weighted MRI to create cortical models, FLAIR and T2-weighted images to exclude brain parenchyma lesion. From our data, there is no subject had significant abnormality in FLAIR and T2-weighted images. This analysis method examines the cortical folding and accurately calculates the cortical thickness. The brain structure was analyzed through segmentation and normalization of the original MR images [16–18], tessellation of the gray and white matter junction [19], and inflation of the folded surface [20], the measurements of the cortical thickness were used to analyze the brain structure.

We used the fully-automated regions of interest (ROIs) which generate by the Freesurfer to define the brain regions and did visually check the output data for quality, registration and segmentation errors [21].

### Statistical analysis

Data are presented as the mean ± standard deviation. A two-tailed independent sample *t* test was performed to compare the patient characteristics and depression scores between the IBS patients and controls. Linear regression was performed to analyze the relationship between the severity of abdominal pain and structural changes in cortical thickness. In addition, to account for the influence of different head sizes and patient ages, a multivariate general linear model was employed and adjusted for age and whole-hemisphere average cortical thickness in order to evaluate the relationships of IBS duration and abdominal pain severity with the cortical thickness between the IBS and control groups.

## Results

### Patient characteristics

We enrolled 29 female IBS patients and 39 female controls from Shin Kong Memorial Hospital. All patients were right-hand dominant. The mean ages of the IBS and control groups were 36.2 ± 7.2 and 37.2 ± 8.3 years, respectively, with no significant age differences noted between the groups (*p* > 0.05; Table 1). The IBS patients had significantly higher depression scores than did the controls (24.47 ± 15.94 vs. 12.33 ± 9.47, *p* < 0.05; Table 1).

**Table 1:** Patient characteristics.

mean±SD	IBS cases (n = 29)	Controls (n = 39)	p value
Age, yr	36.2±7.2	37.2±8.3	0.64
Height, cm	158.5±4.6	159.9±4.2	0.19
Weight, kg	53.8±6.2	55.9±7.2	0.20
Waist, cm	70.2±6.7	71.8±6.9	0.33
Depression scale	25.0±15.9	12.3±9.5	0.0006

#### Differences in cortical thickness between the IBS and control groups

**Fig 1:**
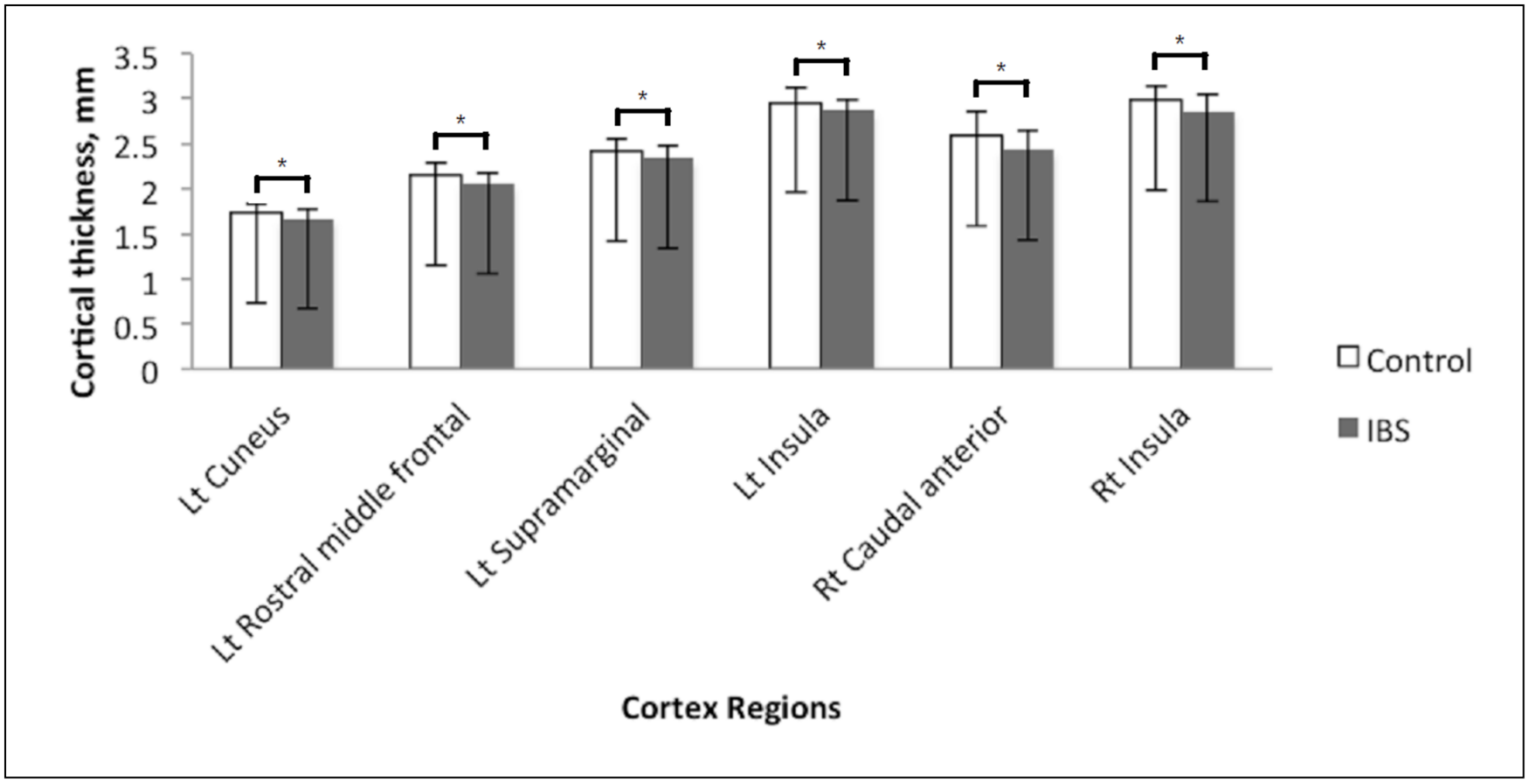
Comparison of thickness in six areas in the cortex between the IBS and control groups.

**Fig 2:**
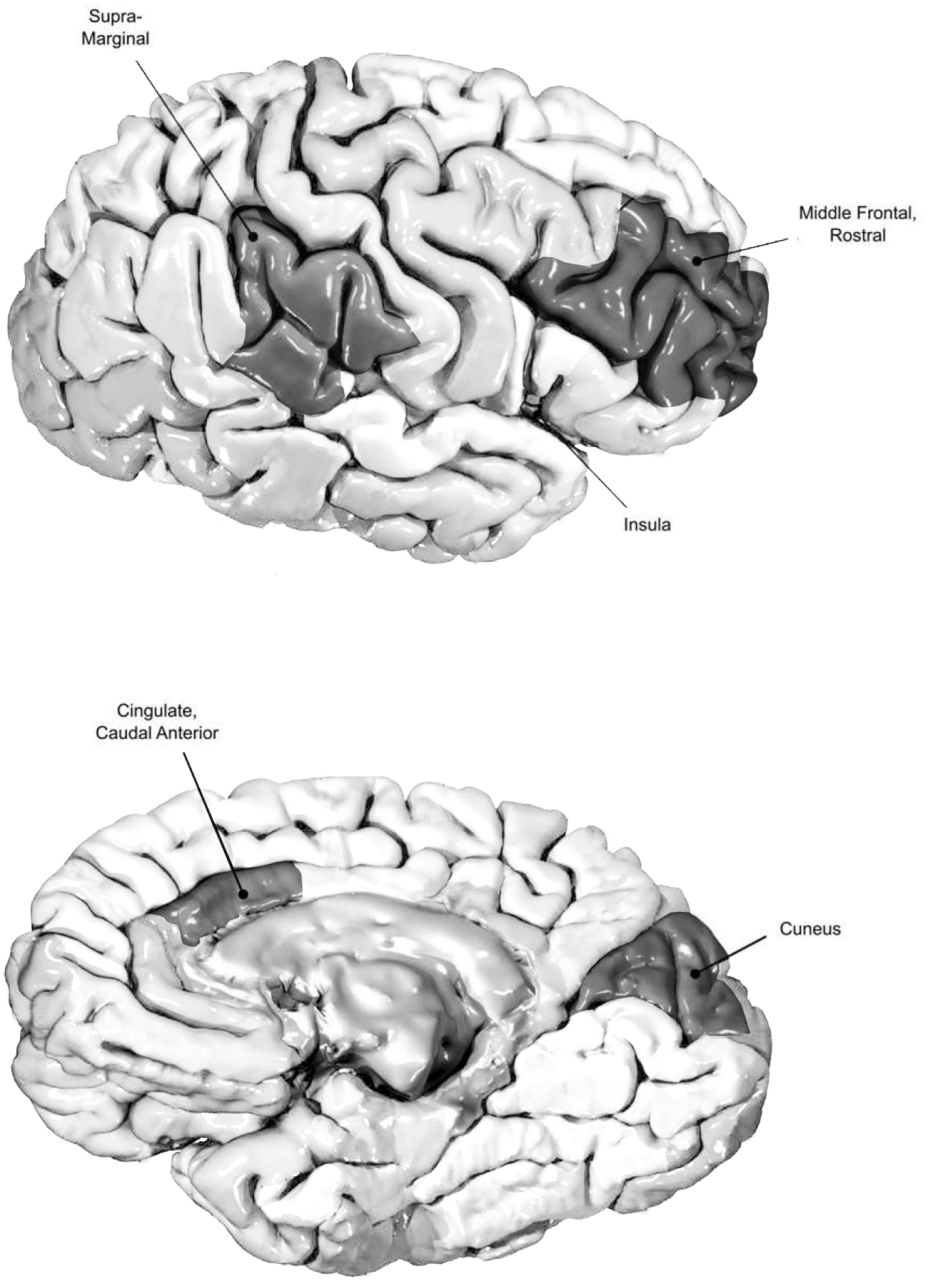
Differences in the affected cortical regions between the IBS and control groups.

Among the 68 brain regions analyzed for cortical thickness, six areas in the cortex exhibited significant differences in thickness between the IBS and control groups. Relative to the controls, the IBS group exhibited gray matter thinning in the left cuneus (1.73 ± 0.10 vs. 1.67 ± 0.10 mm; *p* = 0.013), left rostral middle frontal cortex (2.15 ± 0.13 vs. 2.06 ± 0.12 mm; *p* = 0.009), left supramarginal cortex (2.42 ± 0.14 vs. 2.34 ± 0.14 mm; *p* = 0.035), right caudal anterior cingulate cortex (ACC; 2.59 ± 0.26 vs. 2.43 ± 0.21 mm; *p* = 0.008), left insula (2.96 ± 0.16 vs. 2.87 ± 0.12 mm; *p* = 0.013), and right insula (2.98 ± 0.16 vs. 2.86 ± 0.19 mm; *p* = 0.005; Figs 1 and 2).

#### Trends of brain cortical thickness relative to IBS duration and abdominal pain scores in IBS patients

We analyzed the severity of abdominal pain in the IBS patients. After adjustment for age, the thickness of the left cuneus, left rostral middle frontal, left supramarginal, left insula and right caudal ACC exhibited significantly negative correlations with the duration of IBS (Table 2). Moreover, the thickness of the left cuneus, left rostral middle frontal cortex, left supramarginal, left insula, right caudal ACC and right insula showed significantly negative correlations with the severity of abdominal pain after adjustment for age (Table 3).

**Table 2:** Trends in brain cortical thickness relative to IBS duration in the IBS patients (n = 29).

	Age adjusted	
	β	p value
Left cuneus thickness	-0.00360	0.0189
Left rostral middle frontal	-0.00395	0.0401
Left supramarginal	-0.00531	0.0117
Left insula	-0.00448	0.0373
Right caudal anterior cingulate	-0.00914	0.0164
Right insula	-0.00461	0.0812

**Table 3:** Trends in brain cortical thickness relative to abdominal pain scores in the IBS patients (n = 29).

	Age adjusted	
	β	p value
Left cuneus thickness	-0.01609	0.0031
Left rostral middle frontal	-0.02052	0.0025
Left supramarginal	-0.01897	0.0122
Left insula	-0.01953	0.0108
Right caudal anterior cingulate	-0.03055	0.0260
Right insula	-0.02593	0.0054

## Discussion

We observed gray matter thinning in numerous regions of the brains of the IBS patients. These regions included the left cuneus, left rostral middle frontal cortex, left supramarginal cortex, right caudal ACC, and bilateral insula. These findings can be explained by May *et al*. [22], who reported that the pathophysiology of thinning in these regions may be caused by a decrease in the content of the cortex, possibly due to decreased cell size, apoptosis of neural cells, death of glia and astrocytes, fewer dendritic spines, and reduced synaptic density.

The brain–gut axis controls the regulation of food digestion. In addition, it is associated with stress and mood disorders related to the symptoms of abdominal pain and altered bowel motility [9]. Cortical thickness is mainly represented by neuron cell bodies in the brain and may consist of neurons of varying size and thinning caused by neuronal loss [23].

The insula is an important region for controlling emotional, pain, and visceral perception [9]; it also regulates the perception of sensation from the body [9]. In our study, after adjustment for age, the right insula and left insula exhibited significant cortical thinning, and the thickness correlated negatively with pain severity and IBS duration, respectively. These findings differ from those of Blankstein *et al*., who observed a positive correlation between insula thickness and pain duration and noted a thinner insular cortex in short-term IBS patients; however, they discovered that longer abdominal pain durations correlated positively with the thickness of the insular cortex [11]. Jiang *et al*. reported a cortical thinning in the insula of IBS female patients, which is similar to our results [24].

In a meta-analysis study of fMRI with rectal inflation, the regions that responded to endogenous pain modulation were activated. In IBS patients, the brain regions for visceral sensation, emotion arousal, and attention processes [10]] in the pregenual ACC and amygdala were more specifically activated. In a functional imaging study, Silverman *et al*. stated that the perception of pain was significantly associated with the activity of the ACC; they demonstrated that in patients with IBS, the ACC failed to respond to the perception of nonpainful stimuli [25]. In our study, thinning was observed in the right caudal ACC, and it was associated with emotional arousal; these findings may be related to increased anxiety, vigilance, and altered autonomic responses in IBS patients. In addition, the Hamilton depression score in the IBS group was higher than that in the control group, and this may also be related to the emotional modulation in the right caudal ACC.

Similar to Blankstein *et al*., we noted gray matter thinning in the right caudal ACC of IBS patients, with a decreased cortex density of the anterior midangulate cortex, which is related to the affective and interoperative processing and modulation of pain sensation [11]. Thinning of the left rostral middle frontal cortex was observed in our study, and the dorsolateral prefrontal cortex was noted to be thinner in the study by Blankstein *et al*. This region has been associated with high pain catastrophizing and is related to descending pain modulation [11].

However, there are still numerous differences between our study and other Western studies. Seminowicz *et al*. used voxel-based morphometry and cortical thickness analysis (SurfStat) to study the brain imaging changes and observed changes in the gray matter of the brain, especially in the regions involved in cognitive and evaluative functioning; however, after the changes associated with anxiety and depression were considered, only decreased gray matter in the prefrontal and posterior parietal cortices remained. According to these findings, we can conclude that pain will not cause changes in the cortical density of the brain, but the cortical density of areas related to the cognitive and attentional modulation of interoceptive information will cause pain amplification in IBS; accordingly, the brain structure may be responsible for the pain in IBS [26]. In our study, gray matter thinning was observed in areas that were involved in pain catastrophizing, and this may explain the abdominal pain mechanism of the IBS patients.

Piche *et al*. performed electrical stimulation of the sural nerve and heterotopic noxious counterstimulation and determined that the insula plays a role in interoception and pain. They also stated that the orbitofrontal cortex was associated with pain inhibition, and a thicker right posterior insula was related to a longer duration of pain in IBS. Because the orbitofrontal cortex receives inputs from visceral sensory areas and provides regulatory outputs to the hypothalamus and midbrain to coordinate visceral function, a thicker right lateral orbitofrontal cortex has been strongly associated with less pain inhibition [27]. Both findings were opposite to our findings, wherein we noted a thinner insula and orbitofrontal cortex.

Similar to the study of Haldane *et al*., who identified a relationship between the cuneus cortex and depression control in bipolar patients [28], we noted that the left cuneus cortex was thinner in IBS patients than in controls, which may be related to the more prominent anxiety and depression characteristics of our patients.

The supramarginal cortex is a part of the parietal cortex and is involved in somatosensory function. Cortical thinning in the left supramarginal area was observed. Jiang *et al*. study demonstrated significantly greater cortical thickness in the S1 region in female patients. The S1 region participates in the sensory discriminative processing of both noxious and innocuous stimuli [24].

The prevalence of depression was higher in the IBS group than in the controls. The connection between the prefrontal gyrus and cingulate cortex is a recognized phenomenon in depressive patients [29]. A meta-analysis demonstrated that patients with depression had a thinner cortex in the insula, orbitofrontal cortex, cingulate cortex, and temporal lobes [30]. We obtained similar findings;, hence, some tricyclic antidepressants may be effective in IBS patients [31]

The reasons for the differences in the findings between the present and previous Western studies remain unclear. However, our study specifically investigated an Asian population, which differs culturally from Western populations. Our sample size was also larger than those of other studies, and females were specifically enrolled in our study, considering that the presentations of female and male IBS patients vary. Because we used FreeSurfer to analyze our MRI data, our results may have been influenced by the different analytic tools, statistical algorithms, and templates used compared with those used by other studies.

## Conclusions

Our findings indicate several specific differences in the cortical thickness of the brain in Asian Chinese female IBS patients compared with healthy individuals, including gray matter thinning in the left cuneus, left rostral middle frontal cortex, left supramarginal cortex, right caudal ACC, and bilateral insula. More frequent depression characteristics were noted in the IBS group. The duration of IBS has correlated negatively with thickness in left cuneus, left rostral middle frontal, left supramarginal, left insula and right caudal ACC. The severity of abdominal pain correlated negatively with the thickness of the involved cortex, including the left cuneus, left rostral middle frontal cortex left supramarginal, right caudal ACC, and bilatera insula. All of these regions were associated with the characteristics of decreased pain inhibition, pain catastrophizing, depression, and anxiety. In our study, significant thinning was observed in the regions involved in pain regulation, and this finding differs from the findings of Western studies; this result may be related to the cultural and sex differences between the current and previous studies.
